# Towards a Theory of Legal Animal Rights: Simple and Fundamental Rights

**DOI:** 10.1093/ojls/gqaa007

**Published:** 2020-06-26

**Authors:** Saskia Stucki

**Keywords:** legal animal rights, simple and fundamental animal rights, theories of rights, animal welfare law

## Abstract

With legal animal rights on the horizon, there is a need for a more systematic theorisation of animal rights as legal rights. This article addresses conceptual, doctrinal and normative issues relating to the nature and foundations of legal animal rights by examining three key questions: can, do and should animals have legal rights? It will show that animals are conceptually possible candidates for rights ascriptions. Moreover, certain ‘animal welfare rights’ could arguably be extracted from existing animal welfare laws, even though these are currently imperfect and weak legal rights at best. Finally, this article introduces the new conceptual vocabulary of *simple* and *fundamental* animal rights, in order to distinguish the weak legal rights that animals may be said to have as a matter of positive law from the kind of strong legal rights that animals ought to have as a matter of future law.

## 1. Introduction: The Need for Legal Animal Rights Theory

Legal animal rights are on the horizon, and there is a need for a legal theory of animal rights—that is, a theory of animal rights as *legal* rights. While there is a diverse body of *moral* and *political* theories of animal rights,^1^ the nature and conceptual foundations of legal animal rights remain remarkably underexplored. As yet, only few and fragmented legal analyses of isolated aspects of animal rights exist.^2^ Other than that, most legal writing in this field operates with a hazily assumed, rudimentary and undifferentiated conception of animal rights—one largely informed by extralegal notions of *moral* animal rights—which tends to obscure rather than illuminate the distinctive nature and features of *legal* animal rights.^3^ A more systematic and nuanced theorisation of legal animal rights is, however, necessary and overdue for two reasons: first, a gradual turn to legal rights in animal rights discourse; and, secondly, the incipient emergence of legal animal rights.

First, while animal rights have originally been framed as moral rights, they are increasingly articulated as potential legal rights. That is, animals’ moral rights are asserted in an ‘ought to be legal rights’-sense (or ‘manifesto sense’)^4^ that demands legal institutionalisation and refers to the corresponding legal rights which animals should ideally have.^5^ A salient reason for transforming moral into legal animal rights is that purely moral rights (which exist prior to and independently of legal validation) do not provide animals with sufficient practical protection, whereas legally recognised rights would be reinforced by the law’s more stringent protection and enforcement mechanisms.^6^ With a view to their (potential) juridification, it seems advisable to rethink and reconstruct animal rights as specifically legal rights, rather than simply importing moral animal rights into the legal domain.^7^

Secondly, and adding urgency to the need for theorisation, legal animal rights are beginning to emerge from existing law. Recently, a few pioneering courts have embarked on a path of judicial creation of animal rights, arriving at them either through a rights-based interpretation of animal welfare legislation or a dynamic interpretation of constitutional (human) rights. Most notably, the Supreme Court of India has extracted a range of animal rights from the Prevention of Cruelty to Animals Act and, by reading them in the light of the Constitution, elevated those statutory rights to the status of fundamental rights.^8^ Furthermore, courts in Argentina^9^ and Colombia^10^ have extended the fundamental right of *habeas corpus*, along with the underlying right to liberty, to captive animals.^11^ These (so far isolated) acts of judicial recognition of animal rights may be read as early manifestations of an incipient formation of legal animal rights. Against this backdrop, there is a pressing practical need for legal animal rights theory, in order to explain and guide the as yet still nascent—and somewhat haphazard—evolution of legal animal rights.

This article seeks to take the first steps towards building a more systematic and nuanced theory of legal animal rights. Navigating the existing theoretical patchwork, the article revisits and connects relevant themes that have so far been addressed only in a scattered or cursory manner, and consolidates them into an overarching framework for legal animal rights. Moreover, tackling the well-known problem of ambiguity and obscurity involved in the generally vague, inconsistent and undifferentiated use of the umbrella term ‘animal rights’, this article brings analytical clarity into the debate by disentangling and unveiling different meanings and facets of legal animal rights.^12^ To this end, the analysis identifies and separates three relevant sets of issues: (i) *conceptual* issues concerning the nature and foundations of legal animal rights, and, more generally, whether animals are the kind of beings who can potentially hold legal rights; (ii) *doctrinal* issues pertaining to existing animal welfare law and whether it confers some legal rights on animals—and, if so, what kind of rights; and (iii) *normative* issues as to why and what kind of legal rights animals ought ideally to have as a matter of future law. These thematic clusters will be addressed through three simple yet key questions: *can*, *do* and *should* animals have legal rights?

Section 2 will show that it is conceptually possible for animals to hold legal rights, and will clarify the formal structure and normative grounds of legal animal rights. Moreover, as section 3 will demonstrate, unwritten animal rights could arguably be extracted from existing animal welfare laws, even though such ‘animal welfare rights’ are currently imperfect and weak legal rights at best. In order to distinguish between these weak legal rights that animals may be said to have as a matter of positive law and the kind of strong legal rights that animals ought to have potentially or ideally, the new conceptual categories of ‘*simple* animal rights’ and ‘*fundamental* animal rights’ will be introduced. Finally, section 4 will explore a range of functional reasons why animals need such strong, fundamental rights as a matter of future law.

## 2. Can Animals Have Legal Rights?

As a preliminary matter, it seems necessary to first address the conceptual issue whether animals potentially *can* have legal rights, irrespective of doctrinal and normative issues as to whether animals *do* in fact have, or *should* have, legal rights. Whether animals are possible or potential right holders—that is, the kind of beings to whom legal rights can be ascribed ‘without conceptual absurdity’^13^—must be determined based on the general nature of rights, which is typically characterised in terms of the structure (or form) and grounds (or ultimate purpose) of rights.^14^ Looking at the idea of animal rights through the lens of general rights theories helps clarify the conceptual foundations of legal animal rights by identifying their possible forms and grounds. The first subsection (A) focusses on two particular forms of conceptually basic rights—claims and liberties—and examines their structural compatibility with animal rights. The second subsection (B) considers the two main competing theories of rights—the will theory and interest theory—and whether, and on what grounds, they can accommodate animals as potential right holders.

### A. The Structure of Legal Animal Rights

The formal structure of rights is generally explicated based on the Hohfeldian typology of rights.^15^ Hohfeld famously noted that the generic term ‘right’ tends to be used indiscriminately to cover ‘any sort of legal advantage’, and distinguished four different types of conceptually basic rights: claims (rights *stricto sensu*), liberties, powers and immunities.^16^ In the following, I will show on the basis of first-order rights^17^—claims and liberties—that legal animal rights are structurally possible, and what such legal relations would consist of.^18^

#### (i) Animal claim rights

To have a right in the strictest sense is ‘to have a claim *to* something and *against* someone’, the claim right necessarily corresponding with that person’s correlative duty towards the right holder to do or not to do something.^19^ This type of right would take the form of animals holding a claim to something against, for example, humans or the state who bear correlative duties to refrain from or perform certain actions. Such legal animal rights could be either *negative rights* (correlative to negative duties) to non-interference or *positive rights* (correlative to positive duties) to the provision of some good or service.^20^ The structure of claim rights seems especially suitable for animals, because these are *passive rights* that concern the conduct of others (the duty bearers) and are simply enjoyed rather than exercised by the right holder.^21^ Claim rights would therefore assign to animals a purely passive position that is specified by the presence and performance of others’ duties towards animals, and would not require any actions by the animals themselves.

#### (ii) Animal liberties

Liberties, by contrast, are *active rights* that concern the right holder’s own conduct. A liberty to engage in or refrain from a certain action is one’s freedom of any contrary duty towards another to eschew or undertake that action, correlative to the no right of another.^22^ On the face of it, the structure of liberties appears to lend itself to animal rights. A liberty right would indicate that an animal is free to engage in or avoid certain behaviours, in the sense of being free from a specific duty to do otherwise. Yet, an obvious objection is that animals are generally incapable of having *any* legal duties.^23^ Given that animals are inevitably in a constant state of ‘no duty’ and thus ‘liberty’,^24^ this seems to render the notion of liberty rights somewhat pointless and redundant in the case of animals, as it would do nothing more than affirm an already and invariably existing natural condition of dutylessness. However, this sort of ‘natural liberty’ is, in and of itself, only a naked liberty, one wholly unprotected against interferences by others.^25^ That is, while animals may have the ‘natural liberty’ of, for example, freedom of movement in the sense of not having (and not being capable of having) a duty not to move around, others do not have a duty *vis-à-vis* the animals not to interfere with the exercise of this liberty by, for example, capturing and caging them.

The added value of turning the ‘natural liberties’ of animals into liberty rights thus lies in the act of transforming unprotected, naked liberties into protected, vested liberties that are shielded from certain modes of interference. Indeed, it seems sensible to think of ‘natural liberties’ as constituting legal rights only when embedded in a ‘protective perimeter’ of claim rights and correlative duties within which such liberties may meaningfully exist and be exercised.^26^ This protective perimeter consists of some general duties (arising not from the liberty right itself, but from other claim rights, such as the right to life and physical integrity) not to engage in ‘at least the cruder forms of interference’, like physical assault or killing, which will preclude most forms of effective interference.^27^ Moreover, liberties may be fortified by specific claim rights and correlative duties strictly designed to protect a particular liberty, such as if the state had a (negative) duty not to build highways that cut across wildlife habitat, or a (positive) duty to build wildlife corridors for such highways, in order to facilitate safe and effective freedom of movement for the animals who live in these fragmented habitats.

#### (iii) Animal rights and duties: correlativity and reciprocity

Lastly, some remarks on the relation between animal rights and duties seem in order. Some commentators hold that animals are unable to possess legal rights based on the influential idea that the capacity for holding rights is inextricably linked with the capacity for bearing duties.^28^ Insofar as animals are not capable of bearing legal duties in any meaningful sense, it follows that animals cannot have legal (claim) rights against other animals, given that those other animals would be incapable of holding the correlative duties. But does this disqualify animals from having legal rights altogether, for instance, against legally competent humans or the state?

While duties are a key component of (first-order) rights—with claim rights necessarily implying the *presence* of a legal duty in others and liberties necessarily implying the *absence* of a legal duty in the right holder^29^—neither of them logically entails that the right holder bear duties *herself*. As Kramer aptly puts it:

Except in the very unusual circumstances where someone holds a right against himself, X’s possession of a legal right does not entail X’s bearing of a legal duty; rather, it entails the bearing of a legal duty by somebody else.^30^

This underscores an important distinction between the conceptually axiomatic *correlativity* of rights and duties—the notion that every claim right necessarily implies a duty—and the idea of a *reciprocity* of rights and duties—the notion that (the capacity for) right holding is conditioned on (the capacity for) duty bearing. While correlativity refers to an existential nexus between a right and a duty held by separate persons within *one and the same legal relation*, reciprocity posits a normative nexus between the right holding and duty bearing of *one and the same person* within separate, logically unrelated legal relations.

The claim that the capacity for right holding is somehow contingent on the right holder’s (logically unrelated) capacity for duty bearing is thus, as Kramer puts it, ‘straightforwardly false’ from a Hohfeldian point of view.^31^ Nevertheless, there may be other, normative reasons (notably underpinned by social contract theory) for asserting that the class of *appropriate* right holders should be limited to those entities that, in addition to being structurally *possible* right holders, are also capable of reciprocating, that is, of being their duty bearers’ duty bearers.^32^ However, such a narrow contractarian framing of right holding should be rejected, not least because it misses the current legal reality.^33^ With a view to legally incompetent humans (eg infants and the mentally incapacitated), contemporary legal systems have manifestly cut the connection between right holding and the capacity for duty bearing.^34^ As Wenar notes, the ‘class of potential right holders has expanded to include duty-less entities’.^35^ Similarly, it would be neither conceptually nor legally apposite to infer from the mere fact that animals do not belong to the class of possible duty bearers that they cannot belong to the class of possible right holders.^36^

### B. The Grounds of Legal Animal Rights

While Hohfeld’s analytical framework is useful to outline the possible forms and composition of legal animal rights, Kelch rightly points out that it remains agnostic as to the normative grounds of potential animal rights.^37^ In this respect, the two dominant theories of rights advance vastly differing accounts of the ultimate purpose of rights and who can potentially have them.^38^ Whereas the idea of animal rights does not resonate well with the will theory, the interest theory quite readily provides a conceptual home for it.

#### (i) Will theory

According to the will theory, the ultimate purpose of rights is to promote and protect some aspect of an individual’s autonomy and self-realisation. A legal right is essentially a ‘legally respected choice’, and the right holder a ‘small scale sovereign’ whose exercise of choice is facilitated by giving her discretionary ‘legal powers of control’ over others’ duties.^39^ The class of potential right holders thus includes only those entities that possess agency and legal competence, which effectively rules out the possibility of animals as right holders, insofar as they lack the sort or degree of agency necessary for the will-theory conception of rights.^40^

However, the fact that animals are not potential right holders under the will theory does not necessarily mean that animals cannot have legal rights altogether. The will theory has attracted abundant criticism for its under-inclusiveness as regards both the class of possible right holders^41^ and the types of rights it can plausibly account for, and thus seems to advance too narrow a conception of rights for it to provide a theoretical foundation for *all* rights.^42^ In particular, it may be noted that the kinds of rights typically contemplated as animal rights are precisely of the sort that generally exceed the explanatory power of the will theory, namely inalienable,^43^ passive,^44^ public-law^45^ rights that protect basic aspects of animals’ (partially historically and socially mediated) vulnerable corporeal existence.^46^ Such rights, then, are best explained on an interest-theoretical basis.

#### (ii) Interest theory

Animal rights theories most commonly ground animal rights in animal interests, and thus naturally gravitate to the interest theory of rights.^47^ According to the interest theory, the ultimate purpose of rights is the protection and advancement of some aspect(s) of an individual’s well-being and interests.^48^ Legal rights are essentially ‘legally-protected interests’ that are of special importance and concern.^49^ With its emphasis on well-being rather than on agency, the interest theory seems more open to the possibility of animal rights from the outset. Indeed, as regards the class of possible right holders, the interest theory does little conceptual filtering beyond requiring that right holders be capable of having interests.^50^ Given that, depending on the underlying definition of ‘interest’, this may cover all animals, plants and, according to some, even inanimate objects, the fairly modest and potentially over-inclusive conceptual criterion of ‘having interests’ is typically complemented by the additional, more restrictive moral criterion of ‘having moral status’.^51^ Pursuant to this limitation, not just *any* being capable of having interests can have rights, but only those whose well-being is not merely of instrumental, but of intrinsic or ‘ultimate value’.^52^

Accordingly, under the interest theory, two conditions must be met for animals to qualify as potential right holders: (i) animals must have interests, (ii) the protection of which is required not merely for ulterior reasons, but for the animals’ own sake, because their well-being is intrinsically valuable. Now, whether animals are capable of having interests in the sense relevant to having rights and whether they have moral status in the sense of inherent or ultimate value is still subject to debate. For example, some have denied that animals possess interests based on an understanding of interests as wants and desires that require complex cognitive abilities such as having beliefs and language.^53^ However, most interest theories opt for a broader understanding of interests in the sense of ‘being in someone’s interest’, meaning that an interest holder can be ‘made better or worse off’ and is able to benefit in some way from protective action.^54^ Typically, though not invariably, the capacity for having interests in this broad sense is bound up with sentience—the capacity for conscious and subjective experiences of pain, suffering and pleasure.^55^ Thus, most interest theorists quite readily accept (sentient) animals as potential right holders, that is, as the kind of beings that are capable of holding legal rights.^56^

More importantly yet for legal purposes, the law already firmly rests on the recognition of (some) animals as beings who possess intrinsically valuable interests. Modern animal welfare legislation cannot be intelligibly explained other than as acknowledging that the animals it protects (i) have morally and legally relevant goods and interests, notably in their welfare, life and physical or mental integrity.^57^ Moreover, it rests on an (implicit or explicit) recognition of those animals as (ii) having moral status in the sense of having intrinsic value. The underlying rationale of modern, non-anthropocentric, ethically motivated animal protection laws is the protection of animals *qua* animals, for their own sake, rather than for instrumental reasons.^58^ Some laws go even further by directly referencing the ‘dignity’ or ‘intrinsic value’ of animals.^59^

It follows that existing animal welfare laws already treat animals as intrinsically valuable holders of some legally relevant interests—and thus as precisely the sorts of beings who possess the qualities that are, under an interest theory of rights, necessary and sufficient for having rights. This, then, prompts the question whether those very laws do not only conceptually allow for *potential* animal rights, but might also give rise to *actual* legal rights for animals.

## 3. Do Animals Have (Simple) Legal Rights?

Notwithstanding that animals *could* have legal rights conceptually, the predominant doctrinal opinion is that, as a matter of positive law, animals *do not* have any, at least not in the sense of proper, legally recognised and claimable rights.^60^ Yet, there is a certain inclination, especially in Anglo-American parlance, to speak—in a rather vague manner—of ‘animal rights’ as if they already exist under current animal welfare legislation. Such talk of existing animal rights is, however, rarely backed up with further substantiations of the underlying claim that animal welfare laws do in fact confer legal rights on animals. In the following, I will examine whether animals’ existing legal protections may be classified as legal rights and, if so, what kind of rights these constitute. The analysis will show (A) that implicit animal rights (hereinafter referred to as ‘animal welfare rights’)^61^ can be extracted from animal welfare laws as correlatives of explicit animal welfare duties, but that this reading remains largely theoretical so far, given that such unwritten animal rights are hardly legally recognised in practice. Moreover, (B) the kind of rights derivable from animal welfare laws are currently at best imperfect and weak rights that do not provide animals with the sort of robust normative protection that is generally associated with legal rights, and typically also expected from legal animal rights *qua* institutionalised moral animal rights. Finally, (C) the new conceptual categories of ‘*simple* animal rights’ and ‘*fundamental* animal rights’ are introduced in order to distinguish, and account for the qualitative differences, between such current, imperfect, weak animal rights and potential, ideal, strong animal rights.

### A. Extracting ‘Animal Welfare Rights’ from Animal Welfare Laws

#### (i) The simple argument from correlativity

Existing animal welfare laws are not framed in the language of rights and do not codify any explicit animal rights. They do, however, impose on people legal duties designed to protect animals—duties that demand some behaviour that is beneficial to the welfare of animals. Some commentators contend that correlative (claim) rights are thereby conferred upon animals as the beneficiaries of such duties.^62^ This view is consistent with, and, indeed, the logical conclusion of, an interest-theoretical analysis.^63^ Recall that rights are essentially legally protected interests of intrinsically valuable individuals, and that a claim right is the ‘position of normative protectedness that consists in being owed a … legal duty’.^64^ Under existing animal welfare laws, some goods of animals are legally protected interests in exactly this sense of ultimately valuable interests that are protected through the imposition of duties on others. However, the inference from existing animal welfare duties to the existence of correlative ‘animal welfare rights’ appears to rely on a somewhat simplistic notion of correlativity, along the lines of ‘where there is a duty there is a right’.^65^ Two objections in particular may be raised against the view that beneficial duties imposed by animal welfare laws are sufficient for creating corresponding legal rights in animals.

First, not every kind of duty entails a correlative right.^66^ While some duties are of an unspecific and general nature, only relational, *directed* duties which are *owed to* rather than merely regarding someone are the correlatives of (claim) rights. Closely related, not everyone who stands to benefit from the performance of another’s duty has a correlative right. According to a standard delimiting criterion, beneficial duties generate rights only in the *intended* beneficiaries of such duties, that is, those who are supposed to benefit from duties designed to protect their interests.^67^ Yet, animal welfare duties, in a contemporary reading, are predominantly understood not as indirect duties *regarding* animals—duties imposed to protect, for example, an owner’s interest in her animal, public sensibilities or the moral character of humans—but as direct duties *owed to* the protected animals themselves.^68^ Moreover, the constitutive purpose of modern animal welfare laws is to protect animals for their own sake. Animals are therefore clearly beneficiaries in a qualified sense, that is, they are not merely accidental or incidental, but the direct and intended primary beneficiaries of animal welfare duties.^69^

Secondly, one may object that an analysis of animal rights as originating from intentionally beneficial duties rests on a conception of rights precisely of the sort which has the stigma of redundancy attached to it. Drawing on Hart, this would appear to cast rights as mere ‘alternative formulation of duties’ and thus ‘no more than a redundant translation of duties … into a terminology of rights’.^70^ Admittedly, as MacCormick aptly puts it:

[To] rest an account of claim rights *solely* on the notion that they exist whenever a legal duty is imposed by a law intended to benefit assignable individuals … is to treat rights as being simply the ‘reflex’ of logically prior duties.^71^

One way of responding to this redundancy problem is to reverse the logical order of rights and duties. On this account, rights are not simply created by (and thus logically posterior to) beneficial duties, but rather the converse: such duties are derived from and generated by (logically antecedent) rights. For example, according to Raz, ‘Rights are grounds of duties in others’ and thus justificationally prior to duties.^72^ However, if rights are understood not just as existentially correlative, but as justificationally prior to duties, identifying intentionally beneficial animal welfare duties as the source of (logically posterior) animal rights will not suffice. In order to accommodate the view that rights are grounds of duties, the aforementioned argument from correlativity needs to be reconsidered and refined.

#### (ii) A qualified argument from correlativity

A refined, and reversed, argument from correlativity must show that animal rights are not merely reflexes *created* by animal welfare duties, but rather the *grounds* for such duties. In other words, positive animal welfare duties must be plausibly explained as some kind of codified reflection, or visible manifestation, of ‘invisible’ background animal rights that give rise to those duties.

This requires further clarification of the notion of a justificational priority of rights over duties. On the face of it, the idea that rights are somehow antecedent to duties appears to be at odds with the Hohfeldian correlativity axiom, which stipulates an existential nexus of mutual entailment between rights and duties—one cannot exist without the other.^73^ Viewed in this light, it seems paradoxical to suggest that rights are causal for the very duties that are simultaneously constitutive of those rights—cause and effect seem to be mutually dependent. Gewirth offers a plausible explanation for this seemingly circular understanding of the relation between rights and duties. He illustrates that the ‘priority of claim rights over duties in the order of justifying purpose or final causality is not antithetical to their being correlative to each other’ by means of an analogy:

Parents are prior to their children in the order of efficient causality, yet the (past or present) existence of parents can be inferred from the existence of children, as well as conversely. Hence, the causal priority of parents to children is compatible with the two groups’ being causally as well as conceptually correlative. The case is similar with rights and duties, except that the ordering relation between them is one of final rather than efficient causality, of justifying purpose rather than bringing-into-existence.^74^

Upon closer examination, this point may be specified even further. To stay with the analogy of (biological)^75^ parents and their children: it is actually the *content* of ‘parents’—a male and a female (who at some point procreate together)—that exists prior to and independently of possibly ensuing ‘children’, whereas this content turns into ‘parents’ only in conjunction with ‘children’. That is, the concepts of ‘parents’ and ‘children’ are mutually entailing, whilst, strictly speaking, it is not ‘parents’, but rather that which will later be called ‘parents’ only once the ‘child’ comes into existence—the pre-existing content—which is antecedent to and causal for ‘children’.

Applied to the issue of rights and duties, this means that it is actually the content of a ‘right’—an interest—that exists prior to and independently of, and is (justificationally) causal for the creation of, a ‘duty’, which, in turn, is constitutive of a ‘right’. The distinction between ‘right’ and its content—an interest—allows the pinpointing of the latter as the reason for, and the former as the concomitant correlative of, a duty imposed to protect the pre-existing interest. It may thus be restated, more precisely, that it is not rights, but the *protected interests* which are grounds of duties. Incidentally, this specification is consistent with Raz’s definition of rights, according to which ‘having a right’ means that an aspect of the right holder’s well-being (her interest) ‘is a sufficient reason for holding some other person(s) to be under a duty’.^76^ Now, the enactment of modern animal welfare laws is in and of itself evidence of the fact that some aspects of animals’ well-being (their interests) are—both temporally and justificationally—causal and a sufficient reason for imposing duties on others. Put differently: animal *interests* are *grounds* of animal welfare *duties*, and this, in turn, is conceptually constitutive of animal *rights*.

In conclusion, existing animal welfare laws could indeed be analysed as comprising unwritten ‘animal welfare rights’ as implicit correlatives of the explicit animal welfare duties imposed on others. The essential feature of legal rules conferring rights is that they specifically aim at protecting individual interests or goods—whether they do so *expressis verbis* or not is irrelevant.^77^ Even so, in order for a right to be an actual (rather than a potential or merely postulated) legal right, it should at least be *legally recognised* (if not claimable and enforceable),^78^ which is determined by the applicable legal rules. In the absence of unequivocal wording, whether a legal norm confers unwritten rights on animals becomes a matter of legal interpretation. While theorists can show that a rights-based approach lies within the bounds of a justifiable interpretation of the law, an actual, valid legal right hardly comes to exist by the mere fact that some theorists claim it exists. For that to happen, it seems instrumental that some public authoritative body, notably a court, recognises it as such. That is, while animals’ existing legal protections may already provide for all the ingredients constitutive of rights, it takes a court to *actualise* this *potential*, by authoritatively interpreting those legal rules as constituting rights of animals. However, because courts, with a few exceptions, have not done so thus far, it seems fair to say that unwritten animal rights are not (yet) legally recognised in practice and remain a mostly theoretical possibility for now.^79^

### B. The Weakness of Current ‘Animal Welfare Rights’

Besides the formal issue of legal recognition, there are substantive reasons for questioning whether the kind of rights extractable from animal welfare laws are really rights at all. This is because current ‘animal welfare rights’ are unusually weak rights that do not afford the sort of strong normative protection that is ordinarily associated with legal rights.^80^ Classifying animals’ existing legal protections as ‘rights’ may thus conflict with the deeply held view that, because they protect interests of special importance, legal rights carry *special normative force*.^81^ This quality is expressed in metaphors of rights as ‘trumps’,^82^ ‘protective fences’,^83^ protective shields or ‘No Trespassing’ signs,^84^ or ‘suits of armor’.^85^ Rights bestow upon individuals and their important interests a particularly robust kind of legal protection against conflicting individual or collective interests, by singling out ‘those interests that are *not to be sacrificed to the utilitarian calculus*’ and ‘whose promotion or protection is to be given *qualitative precedence* over the social calculus of interests generally’.^86^ Current ‘animal welfare rights’, by contrast, provide an atypically weak form of legal protection, notably for two reasons: because they protect interests of secondary importance or because they are easily overridden.

In order to illustrate this, consider the kind of rights that can be extracted from current animal welfare laws. Given that these are the correlatives of existing animal welfare duties, the substance of these rights must mirror the content laid down in the respective legal norms. This extraction method produces, first, a rather odd subgroup of ‘animal welfare rights’ that have a narrow substantive scope protecting highly specific, secondary interests, such as a (relative) right to be slaughtered with prior stunning,^87^ an (absolute) right that experiments involving ‘serious injuries that may cause severe pain shall not be carried out without anaesthesia’^88^ or a right of chicks to be killed by fast-acting methods, such as homogenisation or gassing, and to not be stacked on top of each other.^89^ The weak and subsidiary character of such rights becomes clearer when placed within the permissive institutional context in which they operate, and when taking into account the more basic interests that are left unprotected.^90^ While these rights may protect certain secondary, derivative interests (such as the interest in being killed *in a painless manner*), they are simultaneously premised on the permissibility of harming the more primary interests at stake (such as the interest in *not being killed* at all). Juxtaposed with the preponderance of suffering and killing that is legally allowed in the first place, phrasing the residual legal protections that animals do receive as ‘rights’ may strike us as misleading.^91^

But then there is a second subgroup of ‘animal welfare rights’, extractable from general animal welfare provisions, that have a broader scope, protecting more basic, primary interests, such as a right to well-being, life,^92^ dignity,^93^ to not suffer unnecessarily,^94^ or against torture and cruel treatment.^95^ Although the object of such rights is of a more fundamental nature, the substantive guarantee of these facially fundamental rights is, to a great extent, eroded by a conspicuously low threshold for permissible infringements.^96^ That is, these rights suffer from a lack of normative force, which manifests in their characteristically *high infringeability* (ie their low resistance to being overridden). Certainly, most rights (whether human or animal) are relative *prima facie* rights that allow for being balanced against conflicting interests and whose *infringement* constitutes a *violation* only when it is not justified, notably in terms of necessity and proportionality.^97^ Taking rights seriously does, however, require certain safeguards ensuring that rights are only overridden by sufficiently important considerations whose weight is proportionate to the interests at stake. As pointed out by Waldron, the idea of rights is seized on as a way of resisting, or at least restricting, the sorts of trade-offs that would be acceptable in an unqualified utilitarian calculus, where ‘important individual interests may end up being traded off against considerations which are intrinsically less important’.^98^ Yet, this is precisely what happens to animals’ *prima facie* protected interests, any of which—irrespective of how important or fundamental they are—may enter the utilitarian calculus, where they typically end up being outweighed by human interests that are comparatively less important or even trivial, notably dietary and fashion preferences, economic profitability, recreation or virtually any other conceivable human interest.^99^

Any ‘animal welfare rights’ that animals may presently be said to have are thus either of the substantively oddly specific, yet rather secondary, kind or, in the case of more fundamental *prima facie* rights, such that are highly infringeable and ‘evaporate in the face of consequential considerations’.^100^ The remaining question is whether these features render animals’ existing legal protections *non-rights* or just particularly unfit or *weak rights*, but rights nonetheless. The answer will depend on whether the quality of special strength, weight or force is considered a conceptually constitutive or merely typical but not essential feature of rights. On the first view, a certain normative force would function as a threshold criterion for determining what counts as a right and for disqualifying those legal protections that may structurally resemble rights but do not meet a minimum weight.^101^ On the second view, the normative force of rights would serve as a variable that defines the particular weight of different types of rights on a spectrum from weak to strong.^102^ To illustrate the intricacies of drawing a clear line between paradigmatically strong rights, weak rights or non-rights based on this criterion, let us return to the analogy with (biological) ‘parents’. In a minimal sense, the concept of ‘parents’ may be essentially defined as ‘biological creators of a child’. Typically, however, a special role as nurturer and caregiver is associated with the concept of ‘parent’. Now, is someone who merely meets the minimal conceptual criterion (by being the biological creator), but not the basic functions attached to the concept (by not giving care), still a ‘parent’? And, if so, to what extent? Are they a full and proper ‘parent’, or merely an imperfect, dysfunctional form of ‘parent’, a bad ‘parent’, but a ‘parent’ nonetheless? Maybe current animal rights are ‘rights’ in a similar sense as an absent, negligent, indifferent biological mother or father who does not assume the role and responsibilities that go along with parenthood is still a ‘parent’. That is, animals’ current legal protections may meet the minimal conceptual criteria for rights, but they do not perform the characteristic normative function of rights. They are, therefore, at best atypically weak and imperfect rights.

### C. The Distinction between Simple and Fundamental Animal Rights

In the light of the aforesaid, *if* one adopts the view that animals’ existing legal protections constitute legal rights—that is, if one concludes that existing animal welfare laws confer legal rights on animals despite a lack of explicit legal enactment or of any coherent judicial recognition of unwritten animal rights, and that the kind of rights extractable from animal welfare law retain their rights character regardless of how weak they are—then an important qualification needs to be made regarding the nature and limits of such ‘animal welfare rights’. In particular, it must be emphasised that this type of legal animal rights falls short of (i) our ordinary understanding of legal rights as particularly robust protections of important interests and (ii) institutionalising the sort of inviolable, basic moral animal rights (along the lines of human rights) that animal rights theorists typically envisage.^103^ It thus seems warranted to separate the kind of imperfect and weak legal rights that animals may be said to have as a matter of positive law from the kind of ideal,^104^ proper, strong fundamental rights that animals potentially ought to have as a matter of future law.

In order to denote and account for the qualitative difference between these two types of legal animal rights, and drawing on similar distinctions as regards the rights of individuals under public and international law,^105^ I propose to use the conceptual categories of *fundamental* animal rights and other, *simple* animal rights. As to the demarcating criteria, we can distinguish between simple and fundamental animal rights based on a combination of two factors: (i) *substance* (fundamentality or non-fundamentality of the protected interests) and (ii) *normative force* (degree of infringeability). Accordingly, simple animal rights can be defined as weak legal rights whose substantive content is of a non-fundamental, ancillary character and/or that lack normative force due to their high infringeability. In contradistinction, fundamental animal rights are strong legal rights along the lines of human rights that are characterised by the cumulative features of substantive fundamentality and normative robustness due to their reduced infringeability.

The ‘animal welfare rights’ derivable from current animal welfare laws are simple animal rights. However, it is worth noting that while the first subtype of substantively non-fundamental ‘animal welfare rights’ belongs to this category irrespective of their infringeability,^106^ the second subtype of substantively fundamental ‘animal welfare rights’ presently falls in this category purely in respect of their characteristically high infringeability. Yet, the latter is a dynamic and changeable feature, insofar as these rights *could* be dealt with, in case of conflict, in a manner whereby they would prove to be more robust. In other words, while the simple animal rights of the second subtype currently lack the normative force of legal rights, they do have the *potential* to become fundamental animal rights. Why animals need such fundamental rights will be explored in the final section.

## 4. Should Animals Have (Fundamental) Legal Rights?

Beyond the imperfect, weak, simple rights that animals may be said to have based on existing animal welfare laws, a final normative question remains with a view to the future law: whether animals ought to have strong legal rights proper. I will focus on fundamental animal rights—such as the right to life, bodily integrity, liberty and freedom from torture—as these correspond best with the kind of ‘ought to be legal rights’ typically alluded to in animal rights discourse. Given the general appeal of rights language, it is not surprising that among animal advocates there is an overall presumption in favour of basic human rights-like animal rights.^107^ However, it is often simply assumed that, rather than elucidated why, legal rights would benefit animals and how this would strengthen their protection. In order to undergird the normative claim that animals *should* have strong legal rights, the following subsections will look at functional reasons why animals *need* such rights.^108^ I will do so through a non-exhaustive exploration of the potential legal advantages and political utility of fundamental animal rights over animals’ current legal protections (be they animal welfare laws or ‘animal welfare rights’).

### A. Procedural Aspect: Standing and Enforceability

Against the backdrop of today’s well-established ‘enforcement gap’ and ‘standing dilemma’,^109^ one of the most practical benefits typically associated with, or expected from, legal animal rights is the facilitation of standing for animals in their own right and, closely related, the availability of more efficient mechanisms for the judicial enforcement of animals’ legal protections.^110^ This is because legal rights usually include the procedural element of having standing to sue, the right to seek redress and powers of enforcement—which would enable animals (represented by legal guardians) to institute legal proceedings in their own right and to assert injuries of their own.^111^ This would also ‘decentralise’ enforcement, that is, it would not be concentrated in the hands (and at the sole discretion) of public authorities, but supplemented by private standing of animals to demand enforcement. Ultimately, such an expanded enforceability could also facilitate incremental legal change by feeding animal rights questions into courts as fora for public deliberation.

However, while standing and enforceability constitute crucial procedural components of *any* effective legal protection of animals, for present purposes, it should be noted that fundamental animal rights (or any legal animal rights) are—albeit maybe conducive—neither necessary nor sufficient to this end. On the one hand, not all legal rights (eg some socio-economic human rights) are necessarily enforceable. Merely conferring legal rights on animals will therefore, in itself, not guarantee sufficient legal protection from a procedural point of view. Rather, fundamental animal rights must encompass certain procedural rights, such as the right to access to justice, in order to make them effectively enforceable. On the other hand, animals or designated animal advocates could simply be granted standing auxiliary to today’s animal welfare laws, which would certainly contribute towards narrowing the enforcement gap.^112^ Yet, standing as such merely offers the *purely procedural* benefit of being able to legally assert and effectively enforce any given legal protections that animals may have, but has no bearing on the *substantive content* of those enforceable protections. Given that the issue is not just one of improving the enforcement of animals’ existing legal protections, but also of substantially improving them, standing alone cannot substitute for strong substantive animal rights. Therefore, animals will ultimately need both strong substantive *and* enforceable rights, which may be best achieved through an interplay of fundamental rights and accompanying procedural guarantees.

### B. Substantive Aspect: Stronger Legal Protection for Important Interests

The aforesaid suggests that the critical function of fundamental animal rights is not procedural in nature; rather, it is to substantively improve and fortify the protection of important animal interests. In particular, fundamental animal rights would strengthen the legal protection of animals on three levels: by establishing an abstract equality of arms, by broadening the scope of protection to include more fundamental substantive guarantees and by raising the burden of justification for infringements.

First of all, fundamental animal rights would create the *structural* preconditions for a level playing field where human and animal interests are both reinforced by equivalent rights, and can thus collide on equal terms. Generally speaking, not all legally recognised interests count equally when balanced against each other, and rights-empowered interests typically take precedence over or are accorded more weight than unqualified competing interests.^113^ At present, the structural makeup of the balancing process governing human–animal conflicts is predisposed towards a prioritisation of human over animal interests. Whereas human interests are buttressed by strong, often fundamental rights (such as economic, religious or property rights), the interests at stake on the animal side, if legally protected at all, enter the utilitarian calculus as unqualified interests that are merely shielded by simple animal welfare laws, or simple rights that evaporate quickly in situations of conflict and do not compare to the sorts of strong rights that reinforce contrary human interests.^114^ In order to achieve some form of abstract equality of arms, animals’ interests need to be shielded by strong legal rights that are a match to humans’ rights. Fundamental animal rights would correct this structural imbalance and set the stage for an equal consideration of interests that is not *a priori* biased in favour of humans’ rights.

Furthermore, as defined above, fundamental animal rights are characterised by both their substantive fundamentality and normative force, and would thus strengthen animals’ legal protection in two crucial respects. On a *substantive level*, fundamental animal rights are grounded in especially important, fundamental interests. Compared to substantively non-fundamental simple animal rights, which provide for narrow substantive guarantees that protect secondary interests, fundamental animal rights would expand the scope of protection to cover a wider array of basic and primary interests. As a result, harming fundamentally important interests of animals—while readily permissible today insofar as such interests are often not legally protected in the first place^115^—would trigger a justification requirement that initially allows those animal interests to enter into a balancing process. For even with fundamental animal rights in play, conflicts between human and animal interests will inevitably continue to exist—albeit at the elevated and abstractly equal level of conflicts of rights—and therefore require some sort of balancing mechanism.^116^

On this *justificatory level*, fundamental animal rights would then demand a special kind and higher burden of justification for infringements.^117^ As demonstrated above, substantively fundamental yet highly infringeable simple animal rights are marked by a conspicuously low threshold for justifiable infringements, and are regularly outweighed by inferior or even trivial human interests. By contrast, the normative force of fundamental animal rights rests on their ability to raise the ‘level of the minimally sufficient justification’.^118^ Modelling these more stringent justification requirements on established principles of fundamental (human) rights adjudication, this would, first, limit the sorts of considerations that constitute a ‘legitimate aim’ which can be balanced against fundamental animal rights. Furthermore, the balancing process must encompass a strict proportionality analysis, comprised of the elements of suitability, necessity and proportionality *stricto sensu*, which would preclude the bulk of the sorts of low-level justifications that are currently sufficient.^119^ This heightened threshold for justifiable infringements, in turn, translates into a decreased infringeability of fundamental animal rights and an increased immunisation of animals’ *prima facie* protected interests against being overridden by conflicting considerations and interests of lesser importance.

Overall, considering this three-layered strengthening of the legal protection of animals’ important interests, fundamental animal rights are likely to set robust limits to the violability and disposability of animals as means to human ends, and to insulate animals from many of the unnecessary and disproportionate inflictions of harm that are presently allowed by law.

### C. Fallback Function: The Role of Rights in Non-ideal Societies

Because contemporary human–animal interactions are, for the most part, detrimental to animals, the latter appear to be in particular need of robust legal protections against humans and society.^120^ Legal rights, as strong (but not impenetrable) shields, provide an instrument well suited for this task, as they operate in a way that singles out and protects important individual goods *against* others and the political community as a whole. For this reason, rights are generally considered an important counter-majoritarian institution, but have also been criticised for their overly individualistic, antagonistic and anti-communitarian framing.^121^ Certainly, it may be debated whether there is a place for the institution of rights in an ideal society—after all, rights are not decrees of nature, but human inventions that are historically and socially contingent.^122^ However, rights are often born from imperfect social conditions, as a ‘response to a failure of social responsibility’^123^ and as corrections of experiences of injustice, or, as Dershowitz puts it: ‘*rights* come from *wrongs*’.^124^ Historical experience suggests that, at least in non-ideal societies, there is a practical need for rights as a safety net—a ‘position of fall-back and security’^125^—that guarantees individuals a minimum degree of protection, in case or because other, less coercive social or moral mechanisms fail to do so.

Yet, as Edmundson rightly points out, this view of rights as backup guarantees does not quite capture the particular need for rights in the case of animals.^126^ It is premised on the existence of a functioning overall social structure that can in some cases, and maybe in the ideal case, substitute for rights. However, unlike many humans, most animals are not embedded in a web of caring, affectionate, benevolent relations with humans to begin with, but rather are caught up in a system of exploitative, instrumental and harmful relations. For the vast majority of animals, it is not enough to say that rights would serve them as fallbacks, because there is nowhere to fall from—by default, animals are already at (or near) the bottom. Accordingly, the concrete need for rights may be more acute in the case of animals, as their function is not merely to complement, but rather to compensate for social and moral responsibility, which is lacking in the first place.^127^ To give a (somewhat exaggerated) example: from the perspective of a critical legal scholar, meta-theorising from his office in the ivory tower, it may seem easier, and even desirable, to intellectually dispense with the abstract notion of rights, whereas for an elephant who is actually hunted down for his ivory tusks, concrete rights may make a very real difference, literally between life and death. Therefore, under the prevailing social conditions, animals need a set of basic rights as a primary ‘pull-up’ rather than as a subsidiary backup—that is, as *compensatory baseline* guarantees rather than as *complementary background* guarantees.

### D. Transformative Function: Rights as ‘Bridges’ between Non-ideal Realities and Normative Ideals

Notwithstanding that animals need fundamental rights, we should not fail to recognise that even the minimum standards such rights are designed to establish and safeguard seem highly ambitious and hardly politically feasible at present. Even a rudimentary protection of fundamental animal rights would require far-ranging changes in our treatment of animals, and may ultimately rule out ‘virtually all existing practices of the animal-use industries’.^128^ Considering how deeply the instrumental and inherently harmful use of animals is woven into the economic and cultural fabric of contemporary societies, and how pervasive animal cruelty is on both an individual and a collective level, the implications of fundamental animal rights indeed seem far removed from present social practices.^129^ This chasm between normative aspirations and the deeply imperfect empirical realities they collide with is not, however, a problem unique to fundamental *animal* rights; rather, it is generally in the nature of fundamental rights—human or animal—to postulate normative goals that remain, to some extent, aspirational and unattainable.^130^ Aspirational rights express commitments to ideals that, even if they may not be fully realisable at the time of their formal recognition, act as a continuous reminder and impulse that stimulates social and legal change towards a more expansive implementation.^131^ In a similar vein, Bilchitz understands fundamental rights as moral ideals that create the pressure for legal institutionalisation and as ‘bridging concepts’ that facilitate the transition from past and present imperfect social realities towards more just societies.^132^

This, then, provides a useful lens for thinking about the aspirational nature and transformative function of fundamental animal rights. Surely, the mere formal recognition of fundamental animal rights will not, by any realistic measure, bring about an instant practical achievement of the ultimate goal of ‘abolishing exploitation and liberating animals from enslavement’.^133^ They do, however, create the legal infrastructure for moving from a non-ideal reality towards more ideal social conditions in which animal rights can be respected. For example, a strong animal right to life would (at least in industrialised societies) preclude most forms of killing animals for food, and would thus certainly conflict with the entrenched practice of eating meat. Yet, while the current social normality of eating animals may make an immediate prohibition of meat production and consumption unrealistic, it is also precisely the reason why animals need a right to life (ie a right not to be eaten), as fundamental rights help to denormalise (formerly) accepted social practices and to establish, internalise and habituate normative boundaries.^134^ Moreover, due to their dynamic nature, fundamental rights can generate successive waves of more stringent and expansive duties over time.^135^ Drawing on Bilchitz, the established concept of ‘progressive realisation’ (originally developed in the context of socio-economic human rights) may offer a helpful legal framework for the gradual practical implementation of animal rights. Accordingly, each fundamental animal right could be seen as comprising a *minimum core* that has to be ensured immediately, coupled with a general *prohibition of retrogressive measures*, and an obligation to progressively move towards a *fuller realisation*.^136^ Therefore, even if fundamental animal rights may currently not be fully realisable, the very act of introducing them into law and committing to them as normative ideals places animals on the ‘legal map’^137^ and will provide a powerful generative basis—a starting point rather than an endpoint^138^—from which a dynamic process towards their more expansive realisation can unfold.

## 5. Conclusion

The question of animal rights has been of long-standing moral concern. More recently, the matter of institutionalising moral animal rights has come to the fore, and attaining legal rights for animals has become an important practical goal of animal advocates. This article started out from the prefatory observation that the process of juridification may already be in its early stages, as judicially recognised animal rights are beginning to emerge from both animal welfare law and human rights law. With legal animal rights on the horizon, the analysis set out to systematically address the arising conceptual, doctrinal and normative issues, in order to provide a theoretical underpinning for this legal development. The article showed that the idea of legal animal rights has a sound basis in both legal theory as well as in existing law. That is, legal animal rights are both conceptually possible and already derivable from current animal welfare laws. However, the analysis has also revealed that the ‘animal welfare rights’ which animals may be said to have as a matter of positive law fall short of providing the sort of strong normative protection that is typically associated with legal rights and that is furthermore expected from legal animal rights *qua* institutionalised moral animal rights. This discrepancy gave rise to a new conceptual distinction between two types of legal animal rights: simple and fundamental animal rights.

While the umbrella term ‘animal rights’ is often used loosely to refer to a wide range of legal protections that the law may grant to animals, distinguishing between simple and fundamental animal rights helps to unveil important differences between what we may currently call ‘legal animal rights’ based on existing animal welfare laws, which are weak legal rights at best, and the kind of strong, fundamental legal rights that animals should have as a matter of future law. This distinction is further conducive to curbing the trivialisation of the language of animal rights, as it allows us to preserve the normative force of fundamental animal rights by separating out weaker rights and classifying them as other, simple animal rights. Lastly, it is interesting to note that, with courts deriving legal animal rights from both animal welfare law and from constitutional, fundamental or human rights law, first prototypes of simple and fundamental animal rights are already discernible in emerging case law. Whereas Christopher Stone once noted that ‘each successive extension of rights to some new entity has been … a bit unthinkable’ throughout legal history,^139^ the findings of this article suggest that we may presently be witnessing a new generation of legal rights in the making—legal animal rights, simple and fundamental.

## Footnotes

1 Seminally, Tom Regan, *The Case for Animal Rights* (University of California Press 1983); Sue Donaldson and Will Kymlicka, *Zoopolis: A Political Theory of Animal Rights* (OUP 2011).

2 See, notably, Matthew H Kramer, ‘Do Animals and Dead People Have Legal Rights?’ (2001) 14 CJLJ 29; Tom L Beauchamp, ‘Rights Theory and Animal Rights’ in Tom L Beauchamp and RG Frey (eds), *The Oxford Handbook of Animal Ethics* (OUP 2011); William A Edmundson, ‘Do Animals Need Rights?’ (2015) 23 Journal of Political Philosophy 345; Gary L Francione, *Animals, Property, and the Law* (first printed 1995, Temple UP 2007) 91ff; Steven M Wise, ‘Hardly a Revolution—The Eligibility of Nonhuman Animals for Dignity-Rights in a Liberal Democracy’ (1998) 22 Vt L Rev 793; Anne Peters, ‘Liberté, Égalité, Animalité: Human-Animal Comparisons in Law’ (2016) 5 TEL 25; Thomas G Kelch, ‘The Role of the Rational and the Emotive in a Theory of Animal Rights’ (1999) 27 BC Envtl Aff L Rev 1.

3 Much legal scholarship deals with animal *rights* in a rather cursory and incidental manner, because it typically focusses on parallel debates that are closely related to, but seen as preceding, the issue of rights. For example, much has been written about the systemic shortcomings of animal *welfare* legislation, which—within the entrenched animal welfare/rights-dualism—has served to undergird calls for shifting towards a *rights*-paradigm for legal protection of animals. Another focal point of legal scholars has been to change the legal status of animals from *property* to *person*, which is taken to be a prerequisite for right holding. Yet, even though legal rights for animals may be the ultimate goal informing these debates, surprisingly little detailed attention has been given to such envisaged legal animal rights per se.

4 Joel Feinberg, *Social Philosophy* (Prentice-Hall 1973) 67.

5 See eg Alasdair Cochrane, *Animal Rights Without Liberation: Applied Ethics and Human Obligations* (Columbia UP 2012) 14–15, 207 (whose ‘account of the moral rights of animals … proposes what the legal rights of animals *ought to be*’); cf Joel Feinberg, ‘In Defence of Moral Rights’ (1992) 12 OJLS 149 (describing this indirect way of referencing legal rights as the ‘“There ought to be a law” theory of moral rights’, 156).

6 As noted by Favre, what is required is ‘that the legal system intervene when personal morals or ethics do not adequately protect animals from human abuse’. David Favre, ‘Integrating Animal Interests into Our Legal System’ (2004) 10 Animal Law Review 87, 88.

7 Even though moral and legal rights are intimately connected (see HLA Hart, ‘Are There Any Natural Rights?’ (1955) 64 Philosophical Review 175, 177), a somewhat distinct (or at least modified and refined) theorisation is warranted because, unlike moral animal rights, legal animal rights are constituted by legal systems, and their existence and scope have to be determined based on the applicable legal rules. As Wise puts it: ‘philosophers argue moral rights; judges decide legal rights’. Steven M Wise, *Drawing the Line: Science and the Case for Animal Rights* (Perseus 2002) 34.

8 Supreme Court of India 7 May 2014, civil appeal no 5387 of 2014 [27] [56] [62ff]; see further Kerala High Court 6 June 2000, AIR 2000 KER 340 (expressing the opinion that ‘legal rights shall not be the exclusive preserve of the humans’, [13]); Delhi High Court 15 May 2015, CRL MC no 2051/2015 [3] [5] (recognizing birds’ ‘fundamental rights to fly in the sky’).

9 Tercer Juzgado de Garantías de Mendoza 3 November 2016, Expte Nro P-72.254/15; this landmark decision was preceded by an *obiter dictum* in Cámara Federal de Casación Penal Buenos Aires, 18 December 2014, SAIJ NV9953 [2] (expressing the view that animals are right holders and should be recognized as legal subjects).

10 Corte Suprema de Justicia 26 July 2017, AHC4806-2017 (MP: Luis Armando Tolosa Villabona). This ruling was later reversed in Corte Suprema de Justicia 16 August 2017, STL12651-2017 (MP: Fernando Castillo Cadena). In January 2020, the Constitutional Court of Colombia decided against granting *habeas corpus* to the animal in question.

11 Similar *habeas corpus* claims on behalf of chimpanzees and elephants, brought by the Nonhuman Rights Project, have not been accepted by US courts. See, notably, *Tommy v Lavery* NY App Div 4 December 2014, Case No 518336.

12 On the ambiguity of the term ‘animal rights’, see eg Will Kymlicka and Sue Donaldson, ‘Rights’ in Lori Gruen (ed), *Critical Terms for Animal Studies* (University of Chicago Press 2018) 320; in using the umbrella term ‘animal rights’ without further specifications, it is often left unclear what exactly is meant by ‘rights’. For example, the term may refer to either moral or legal animal rights—or both. Furthermore, in a broad sense, ‘animal rights’ sometimes refers to any kind of normative protection for animals, whereas in a narrow sense, it is often reserved for particularly important and inviolable, human rights-like animal rights. Moreover, some speak of ‘animal rights’ as if they already existed as a matter of positive law, while others use the same term in a ‘manifesto sense’, to refer to potential, ideal rights.

13 Joel Feinberg, ‘Human Duties and Animal Rights’ in Clare Palmer (ed), *Animal Rights* (Routledge 2008) 409; the class of potential right holders comprises ‘any being that is capable of holding legal rights, whether or not he/she/it actually holds such rights’. Kramer, ‘Do Animals and Dead People Have Legal Rights?’ (n 2) 29.

14 See generally Alon Harel, ‘Theories of Rights’ in Martin P Golding and William A Edmundson (eds), *Philosophy of Law and Legal Theory* (Blackwell 2005) 191ff.

15 Wesley Newcomb Hohfeld, ‘Some Fundamental Legal Conceptions as Applied in Judicial Reasoning’ (1913) 23 Yale LJ 16; Wesley Newcomb Hohfeld, ‘Fundamental Legal Conceptions as Applied in Judicial Reasoning’ (1917) 26 Yale LJ 710.

16 See Hohfeld, ‘Fundamental Legal Conceptions’ (n 15) 717; these Hohfeldian incidents of rights are merely ‘atomic’ units, whereas many common rights are complex aggregates, clusters or ‘molecular rights’ consisting of combinations thereof. ibid 746; Leif Wenar, ‘The Nature of Rights’ (2005) 33 Philosophy & Public Affairs 223, 225, 234.

17 First-order rights (claims and liberties) directly concern someone’s actual rather than normative conduct, whereas powers and immunities are second-order rights (‘meta-rights’) that concern other legal relations; by prioritising, for the sake of this analysis, first-order rights regarding (in)actions of and towards animals, this is not to say that second-order rights are not important to accompany and bolster the first-order rights of animals. For instance, just as many complex (eg fundamental) rights contain immunities, that is, the freedom from the legal power of another (the disability bearer) to change the immunity holder’s rights, animals’ claims and liberties may be bolstered by immunity rights that protect those first-order rights from being altered, notably voided, by others. For example, one of the most basic rights frequently discussed for animals, the ‘right not to be property’ (Gary L Francione, *Introduction to Animal Rights: Your Child or the Dog?* (first printed 2000, Temple UP 2007) 93ff), may be explained as an immunity that would strip away the legal powers that currently go along with the state of legal disposability entailed by animals’ property status, and would thus disable human ‘owners’ to decide over animals’ rights. As passive rights, immunities are quite easily conceivable as animal rights, because they are specified by reference to the correlative position, that is, by what the person disabled by the animal’s immunity right cannot legally do (see generally Matthew H Kramer, ‘Rights Without Trimmings’ in Matthew H Kramer, NE Simmonds and Hillel Steiner, *A Debate Over Rights: Philosophical Enquiries* (OUP 1998) 22). By contrast, a power refers to one’s control over a given legal relation and entails one’s normative ability to alter another’s legal position (see Hohfeld, ‘Some Fundamental Legal Conceptions’ (n 15) 55). *Prima facie*, powers may thus seem ill-suited for animals. This is because, unlike passive second-order rights (immunities), powers are active rights that have to be exercised rather than merely enjoyed and, unlike first-order active rights (liberties), powers concern the exercise of legal rather than factual actions and thus require legal rather than mere practical or behavioural agency. Notwithstanding, it may be argued that animals, not unlike children, could hold legal powers (eg powers of enforcement) that are exercisable through human proxies (cf Visa AJ Kurki, ‘Legal Competence and Legal Power’ in Mark McBride (ed), *New Essays on the Nature of Rights* (Hart Publishing 2017) 46).

18 For a discussion of Hohfeldian theory in the context of animal rights, see also Wise, ‘Hardly a Revolution’ (n 2) 799ff; Francione, *Animals, Property, and the Law* (n 2) 96–7; Kelch, ‘The Role of the Rational’ (n 2) 6ff.

19 Joel Feinberg, ‘The Rights of Animals and Unborn Generations’ in Joel Feinberg, *Rights, Justice, and the Bounds of Liberty: Essays in Social Philosophy* (Princeton UP 1980) 159; Hohfeld, ‘Some Fundamental Legal Conceptions’ (n 15) 55.

20 So far, animal rights theory has largely focussed on negative rights. See critically Donaldson and Kymlicka (n 1) 5ff, 49ff.

21 cf Wenar, ‘The Nature of Rights’ (n 16) 233.

22 See Hohfeld, ‘Some Fundamental Legal Conceptions’ (n 15) 55; Kramer, ‘Rights Without Trimmings’ (n 17) 10.

23 See eg Feinberg, ‘The Rights of Animals and Unborn Generations’ (n 19) 162; but see Kramer, ‘Do Animals and Dead People Have Legal Rights?’ (n 2) 41–2 (arguing that it would not be impossible, though ‘cruel and perhaps silly’, to impose legal duties on animals).

24 A ‘liberty’ is the negation of ‘duty’ and may thus be redescribed as ‘no-duty’.

25 On the distinction between naked and vested liberties, see HLA Hart, ‘Legal Rights’ in HLA Hart, *Essays on Bentham: Studies in Jurisprudence and Political Theory* (OUP 1982) 172.

26 Hart, ‘Legal Rights’ (n 25) 171, 173.

27 Hart, ‘Legal Rights’ (n 25) 171.

28 eg Richard L Cupp, ‘Children, Chimps, and Rights: Arguments from “Marginal” Cases’ (2013) 45 Ariz St LJ 1; see also Christine M Korsgaard, *Fellow Creatures: Our Obligations to the Other Animals* (OUP 2018) 116ff.

29 See David Lyons, ‘Rights, Claimants, and Beneficiaries’ (1969) 6 American Philosophical Quarterly 173, 173–4.

30 Kramer, ‘Do Animals and Dead People Have Legal Rights?’ (n 2) 42.

31 Kramer, ‘Do Animals and Dead People Have Legal Rights?’ (n 2) 42.

32 In this vein, *Tommy v Lavery* NY App Div 4 December 2014, Case No 518336, p 4, 6; but see critically New York Court of Appeals, *Tommy v Lavery* and *Kiko v Presti* decision of 8 May 2018, motion no 2018-268, concurring opinion Judge Fahey.

33 For example, the Supreme Court of Colombia explicitly departed from this reciprocity paradigm and held that animals are right holders but not duty bearers. Corte Suprema de Justicia 26 July 2017, AHC4806-2017 (MP: Luis Armando Tolosa Villabona), 14ff; for a refutation of the contractarian reciprocity argument, see also Brief for Philosophers as Amici Curiae Supporting Petitioner-Appellant, *Nonhuman Rights Project v Lavery* 2018 NY Slip Op 03309 (2018) (Nos 162358/15 and 150149/16), 14ff.

34 See Peters (n 2) 45–6; David Bilchitz, ‘Moving Beyond Arbitrariness: The Legal Personhood and Dignity of Non-Human Animals’ (2009) 25 SAJHR 38, 42–3; Feinberg, ‘The Rights of Animals and Unborn Generations’ (n 19) 163; but see *Tommy v Lavery* NY App Div 4 December 2014, Case No 518336, 5.

35 Leif Wenar, ‘The Nature of Claim Rights’ (2013) 123 Ethics 202, 207.

36 See Kramer, ‘Do Animals and Dead People Have Legal Rights?’ (n 2) 43.

37 See Kelch, ‘The Role of the Rational’ (n 2) 9.

38 For an overview, see generally Matthew H Kramer, NE Simmonds and Hillel Steiner, *A Debate Over Rights: Philosophical Enquiries* (OUP 1998).

39 Hart, ‘Legal Rights’ (n 25) 183, 188–9.

40 See Kramer, ‘Do Animals and Dead People Have Legal Rights?’ (n 2) 30; Hart, ‘Legal Rights’ (n 25) 185.

41 A problematic corollary of the will theory is its conceptual awkwardness, or inability, to accommodate as right holders not just non-human but also human non-agents, such as infants and the mentally incapacitated. As noted by Hart, ‘Are There Any Natural Rights?’ (n 7) 181, the will conception of rights ‘should incline us not to extend to animals and babies … the notion of a right’; see also Kramer, ‘Rights Without Trimmings’ (n 17) 69.

42 As pointed out by van Duffel, neither the will theory nor the interest theory may be a ‘plausible candidate for a comprehensive theory of rights’, and it may be best to assume that both theories simply attempt to capture the essence of different kinds of rights. See Siegfried van Duffel, ‘The Nature of Rights Debate Rests on a Mistake’ (2012) 93 Pacific Philosophical Quarterly 104, 105, 117 *et passim*.

43 Under the will theory, inalienable rights are not ‘rights’ by definition, as they precisely preclude the right holder’s power to waive the correlative duties. See DN MacCormick, ‘Rights in Legislation’ in PMS Hacker and J Raz (eds), *Law, Morality, and Society: Essays in Honour of HLA Hart* (OUP 1977) 198f; Kramer, ‘Rights Without Trimmings’ (n 17) 73.

44 The will theory is primarily modelled on active rights (liberties and powers) that directly facilitate individual autonomy and choice, but is less conclusive with regard to passive rights (claims and immunities) which do not involve any action or exercise of choice by the right holder herself. cf Harel (n 14) 194–5.

45 Hart, ‘Legal Rights’ (n 25) 190, conceded that the will theory does not provide a sufficient analysis of constitutionally guaranteed fundamental rights; legal animal rights, by contrast, are most intelligibly explained as public-law rights held primarily against the state which has correlative duties to respect and protect.

46 The will theory appears to limit the purpose of rights protection to a narrow aspect of human nature—the active, engaging and self-determining side—while ignoring the passive, vulnerable and needy side. Autonomy is certainly an important good deserving of normative protection, but it is hardly the only such good. See Jeremy Waldron, ‘Introduction’ in Jeremy Waldron (ed), *Theories of Rights* (OUP 1984) 11; MacCormick, ‘Rights in Legislation’ (n 43) 197, 208.

47 See Kelch, ‘The Role of the Rational’ (n 2) 10ff; for an interest-based approach to animal rights, see eg Feinberg, ‘The Rights of Animals and Unborn Generations’ (n 19); Cochrane (n 5) 19ff.

48 Kramer, ‘Do Animals and Dead People Have Legal Rights?’ (n 2) 29; MacCormick, ‘Rights in Legislation’ (n 43) 192.

49 J Raz, ‘Legal Rights’ (1984) 4 OJLS 1, 12; Waldron, ‘Introduction’ (n 46) 12, 14.

50 See William A Edmundson, *An Introduction to Rights* (2nd edn, CUP 2012) 97; Joseph Raz, *The Morality of Freedom* (Clarendon Press 1986) 176; Feinberg, ‘The Rights of Animals and Unborn Generations’ (n 19) 167.

51 See Kramer, ‘Do Animals and Dead People Have Legal Rights?’ (n 2) 33ff, 39.

52 Raz, *The Morality of Freedom* (n 50) 166, 177ff; see also Neil MacCormick, ‘Children’s Rights: A Test-Case for Theories of Right’ in Neil MacCormick, *Legal Right and Social Democracy: Essays in Legal and Political Philosophy* (OUP 1982) 159–60.

53 See RG Frey, *Interests and Rights: The Case Against Animals* (OUP 1980) 78ff; HJ McCloskey, ‘Rights’ (1965) 15 The Philosophical Quarterly 115, 126; but see Tom Regan, ‘McCloskey on Why Animals Cannot Have Rights’ (1976) 26 The Philosophical Quarterly 251.

54 Harel (n 14) 195; Kramer, ‘Do Animals and Dead People Have Legal Rights?’ (n 2) 33.

55 See eg Feinberg, ‘The Rights of Animals and Unborn Generations’ (n 19) 166; Kramer, ‘Do Animals and Dead People Have Legal Rights?’ (n 2) 39–40; Visa AJ Kurki, ‘Why Things Can Hold Rights: Reconceptualizing the Legal Person’ in Visa AJ Kurki and Tomasz Pietrzykowski (eds), *Legal Personhood: Animals, Artificial Intelligence and the Unborn* (Springer 2017) 79–80.

56 See eg Wenar, ‘The Nature of Claim Rights’ (n 35) 207, 227; Kramer, ‘Do Animals and Dead People Have Legal Rights?’ (n 2) 54; Feinberg, ‘The Rights of Animals and Unborn Generations’ (n 19) 166.

57 See also Kurki, ‘Why Things Can Hold Rights’ (n 55) 80.

58 See Thomas G Kelch, ‘A Short History of (Mostly) Western Animal Law: Part II’ (2013) 19 Animal Law Review 347, 348ff; Bilchitz, ‘Moving Beyond Arbitrariness’ (n 34) 44ff; in this vein, the Constitutional Court of South Africa (8 December 2016, CCT 1/16 [57]) noted that ‘the rationale behind protecting animal welfare has shifted from merely safeguarding the moral status of humans to *placing intrinsic value on animals as individuals*’ (emphasis added); the well-established German concept of ‘ethischer Tierschutz’ expresses this non-anthropocentric, ethical thrust of animal welfare law. See Margot Michel, ‘Law and Animals: An Introduction to Current European Animal Protection Legislation’ in Anne Peters, Saskia Stucki and Livia Boscardin (eds), *Animal Law: Reform or Revolution?* (Schulthess 2015) 91–2.

59 1999 Federal Constitution (Bundesverfassung) (CH), Article 120(2) and 2005 Animal Welfare Act (Tierschutzgesetz) (CH), Article 1 and 3(a); 2010 Animal Welfare Act (Tierschutzgesetz) (LI), Article 1; 2018 Animal Welfare Act (Loi sur la protection des animaux) (LU), Article 1; 1977 Experiments on Animals Act (Wet op de dierproeven) (NL), Article 1a; European Parliament and Council Directive 2010/63/EU of 22 September 2010 on the protection of animals used for scientific purposes [2010] OJ L276/33, Recital 12.

60 See eg Steven M Wise, ‘Legal Rights for Nonhuman Animals: The Case for Chimpanzees and Bonobos’ (1996) 2 Animal Law Review 179, 179; Richard A Epstein, ‘Animals as Objects, or Subjects, of Rights’ in Cass R Sunstein and Martha C Nussbaum (eds), *Animal Rights: Current Debates and New Directions* (OUP 2005) 144ff; Francione, *Animals, Property, and the Law* (n 2) 91ff; Kelch, ‘The Role of the Rational’ (n 2) 18; Court of Appeal of Alberta, *Reece v Edmonton (City)*, 2011 ABCA 238 [6]; *Herrmann v Germany* App no 9300/07 (ECtHR, 26 June 2012), separate opinion of Judge Pinto de Albuquerque, 38; *Noah v Attorney General* HCJ 9232/01 [2002–2003] IsrLR 215, 225, 232, 253.

61 This type of current legal animal rights will be called ‘animal welfare rights’ in order to indicate their origin in current animal welfare laws.

62 See eg Cass R Sunstein, ‘Standing for Animals (with Notes on Animal Rights)’ (2000) 47 UCLA Law Review 1333 (claiming that current animal welfare law creates ‘a robust set of animal rights’ or even ‘an incipient bill of rights for animals’. ibid 1334, 1336); Bilchitz, ‘Moving Beyond Arbitrariness’ (n 34) 43ff, 48–9 (concluding that ‘the existing statutory framework can already be seen to confer certain legal rights upon animals’: 50 fn 61); Jerrold Tannenbaum, ‘Animals and the Law: Property, Cruelty, Rights’ (1995) 62 Social Research 539, 581; Beauchamp (n 2) 207; Wise, ‘Hardly a Revolution’ (n 2) 910ff; this view was endorsed by the Supreme Court of India 7 May 2014, civil appeal no 5387 of 2014 [27] (stating that the Prevention of Cruelty to Animals Act ‘deals with duties of persons having charge of animals, which is mandatory in nature and hence confer corresponding rights on animals’).

63 See eg Joel Feinberg, ‘Human Duties and Animal Rights’ in Feinberg, *Rights, Justice, and the Bounds of Liberty* (n 19) 193–4 *et passim*; Kramer, ‘Do Animals and Dead People Have Legal Rights?’ (n 2) 54; Wenar, ‘The Nature of Claim Rights’ (n 35) 218, 220; Visa AJ Kurki, *A Theory of Legal Personhood* (OUP 2019) 62–5.

64 Matthew H Kramer, ‘Legal and Moral Obligation’ in Martin P Golding and William A Edmundson (eds), *The Blackwell Guide to the Philosophy of Law and Legal Theory* (Blackwell 2005) 188.

65 eg, for Sunstein correlativity seems to run both ways: ‘Not only do rights create duties, but the imposition of a duty also serves to create a right.’ Cass R Sunstein, ‘Rights and Their Critics’ (1995) 70 Notre Dame L Rev 727, 746.

66 On this objection, see also Kelch, ‘The Role of the Rational’ (n 2) 8–9.

67 See Lyons (n 29) 176; Waldron, ‘Introduction’ (n 46) 10; critically Kramer, ‘Rights Without Trimmings’ (n 17) 85ff; Visa AJ Kurki, ‘Rights, Harming and Wronging: A Restatement of the Interest Theory’ (2018) 38 OJLS 430, 436ff.

68 See eg Beauchamp (n 2) 207; Feinberg, ‘The Rights of Animals and Unborn Generations’ (n 19) 161–2, 166; Bilchitz, ‘Moving Beyond Arbitrariness’ (n 34) 45–6; in this vein, a German high court held that, based on the criminal law justification of necessity (‘rechtfertigender Notstand’), private persons may be authorised to defend the legally protected goods of animals *on behalf of* the animals, independently of or even against the interests of their owners. OLG Naumburg, judgment of 22 February 2018, case no 2 Rv 157/17, recital II; on why animals need directed rather than indirect duties, see Edmundson, ‘Do Animals Need Rights?’ (n 2) 350ff.

69 See also Francione, *Animals, Property, and the Law* (n 2) 100.

70 Hart, ‘Legal Rights’ (n 25) 181–2, 190.

71 MacCormick, ‘Rights in Legislation’ (n 43) 199.

72 Raz, *The Morality of Freedom* (n 50) 167, 170f; see also Alan Gewirth, ‘Introduction’ in Alan Gewirth, *Human Rights: Essays on Justification and Applications* (University of Chicago Press 1982) 14.

73 See Kramer, ‘Rights Without Trimmings’ (n 17) 40.

74 Gewirth (n 72) 14.

75 For the sake of the argument, I am only referring to *biological* parents.

76 Raz, *The Morality of Freedom* (n 50) 166, 180–1.

77 See MacCormick, ‘Rights in Legislation’ (n 43) 191–2; Raz, ‘Legal Rights’ (n 49) 13–14.

78 According to some scholars, legal rights exist only when they are enforceable. See eg Ronald Dworkin, *Justice for Hedgehogs* (Harvard UP 2011) 405–6 (stating that legal rights are only those that the right holder is entitled to enforce on demand in directly available adjudicative processes).

79 A significant practical hurdle to the legal recognition of animal rights is that in virtually any legal order, animals are legal objects rather than legal persons. Because legal personhood and right holding are generally thought to be inextricably linked, many jurists refrain from calling the existing legal protections of animals ‘rights’. See critically Kurki, ‘Why Things Can Hold Rights’ (n 55) 71, 85–6.

80 See generally Francione, *Animals, Property, and the Law* (n 2) 91ff.

81 On this, see Kai Möller, ‘Proportionality and Rights Inflation’ in Grant Huscroft, Bradley W Miller and Grégoire Webber, *Proportionality and the Rule of Law: Rights, Justification, Reasoning* (CUP 2014) 166; Harel (n 14) 197ff; Waldron, ‘Introduction’ (n 46) 14ff.

82 Ronald Dworkin, ‘Rights as Trumps’ in Waldron, *Theories of Rights* (n 46) 153.

83 Bernard E Rollin, ‘The Legal and Moral Bases of Animal Rights’ in HB Miller and WH Willliams (eds), *Ethics and Animals* (Humana Press 1983) 106.

84 Tom Regan, ‘The Day May Come: Legal Rights for Animals’ (2004) 10 Animal Law Review 11, 15–16.

85 Frederick Schauer, ‘A Comment on the Structure of Rights’ (1993) 27 Ga L Rev 415, 429 *et passim*.

86 Jeremy Waldron, ‘Rights in Conflict’ in Jeremy Waldron, *Liberal Rights: Collected Papers 1981–1991* (CUP 1993) 209, 215–16 (emphasis added); see also Frederick Schauer, ‘Rights, Constitutions and the Perils of Panglossianism’ (2018) 38 OJLS 635, 637.

87 Correlative to Council Regulation (EC) 1099/2009 of 24 September 2009 on the protection of animals at the time of killing [2009] OJ L303/1, Article 4 and Annex I.

88 Correlative to European Parliament and Council Directive 2010/63/EU of 22 September 2010 on the protection of animals used for scientific purposes [2010] OJ L276/33, Article 14(1)(2).

89 Correlative to 2008 Animal Welfare Ordinance (Tierschutzverordnung) (CH), Article 178a(3).

90 The permissive character of animal welfare law was highlighted by the Israeli High Court of Justice in a case concerning the force-feeding of geese. Commenting on the ‘problematic’ regulatory language, it noted that the stated ‘purpose of the Regulations is “to prevent the geese’s suffering.” Clearly these regulations do not prevent suffering; at best they minimize, to some extent, the suffering caused’. *Noah v Attorney General* (n 60) 234–5. See also Shai Lavi, ‘Humane Killing and the Ethics of the Secular: Regulating the Death Penalty, Euthanasia, and Animal Slaughter’ (2014) 4 UC Irvine Law Review 297, 321 (noting the disparity between ‘the resolution to overcome pain and suffering, which exists side-by-side with inhumane conditions that remain unchallenged and are often taken for granted’).

91 As MacCormick, ‘Children’s Rights’ (n 52) 159, has succinctly put it: ‘Consider the oddity of saying that turkeys have a right to be well fed in order to be fat for the Christmas table’; this is not to minimise the importance of existing animal welfare protections. Even though they are insufficient and weak compared to proper legal rights, that does not mean that they are insignificant. See, on this point, Regina Binder, ‘Animal Welfare Regulation: Shortcomings, Requirements, Perspectives’ in Anne Peters, Saskia Stucki and Livia Boscardin (eds), *Animal Law: Reform or Revolution?* (Schulthess 2015) 83.

92 eg correlative to 1972 Animal Welfare Act (Tierschutzgesetz) (DE), § 1 and 17(1).

93 eg correlative to 2005 Animal Welfare Act (Tierschutzgesetz) (CH), Article 1 and 26(1)(a).

94 eg derived from Animal Welfare Act 2006 (UK), s 4.

95 See eg Supreme Court of India 7 May 2014, civil appeal no 5387 of 2014 [62] (extracting from animal welfare law, *inter alia*, the right to life, to food and shelter, to dignity and fair treatment, and against torture); similarly, Court of Appeal of Alberta, *Reece v Edmonton (City)*, 2011 ABCA 238, dissenting opinion Justice Fraser [43].

96 For example, the *prima facie* right to be free from unnecessary pain and suffering is, in effect, rendered void if virtually any kind of instrumental interest in using animals is deemed necessary and a sufficient justification for its infringement.

97 See Edmundson, ‘Do Animals Need Rights?’ (n 2) 346; Harel (n 14) 198; Laurence H Tribe, ‘Ten Lessons Our Constitutional Experience Can Teach Us About the Puzzle of Animal Rights: The Work of Steven M Wise’ (2001) 7 Animal Law Review 1, 2.

98 See Waldron, ‘Rights in Conflict’ (n 86) 209–11.

99 See Francione, *Animals, Property, and the Law* (n 2) 17ff, 109.

100 Francione, *Animals, Property, and the Law* (n 2) 114.

101 For Schauer, a certain normative force seems to be constitutive of the concept of rights. He argues that a right exists only insofar as an interest is protected against the sorts of low-level justifications that would otherwise be sufficient to restrict the interest if it were not protected by the right. See Schauer, ‘A Comment on the Structure of Rights’ (n 85) 430 *et passim*.

102 In this vein, Sunstein holds that animal welfare laws ‘protect a form of animal rights, and there is nothing in the notion of rights or welfare that calls for much, or little, protection of the relevant interests’. Sunstein, ‘Standing for Animals’ (n 62) 1335.

103 On the universal basic rights of animals, see eg Donaldson and Kymlicka (n 1) 19ff.

104 ‘Ideal right’ in the sense of ‘what *ought* to be a positive … right, and would be so in a better or ideal legal system’. Feinberg, *Social Philosophy* (n 4) 84.

105 In domestic public law, fundamental or constitutional rights are distinguished from other, simple public (eg administrative) law rights. Likewise, in international law, human rights can be distinguished from other, simple or ordinary international individual rights. See Anne Peters, *Beyond Human Rights: The Legal Status of the Individual in International Law* (CUP 2016) 436ff.

106 Indeed, substantively non-fundamental simple animal rights may be quite resistant to being overridden, and may sometimes even be absolute (non-infringeable) rights.

107 Nonetheless, the usefulness of legal rights is not undisputed within the animal advocacy movement. For an overview of some pragmatic and principled objections against animal *rights*, see Kymlicka and Donaldson (n 12) 325ff.

108 See generally Edmundson, ‘Do Animals Need Rights?’ (n 2); Peters (n 2) 46ff.

109 Today, animals’ legal protections remain pervasively under-enforced by the competent public authorities as well as practically unenforceable by the affected animals or their human representatives for lack of standing. See eg Sunstein, ‘Standing for Animals’ (n 62) 1334ff; Tribe (n 97) 3.

110 The link between rights and the legal-operational advantage of standing was famously highlighted by Christopher D Stone, ‘Should Trees Have Standing? Toward Legal Rights for Natural Objects’ (1972) 45 S Cal L Rev 450; see further Cass R Sunstein, ‘Can Animals Sue?’ in Cass R Sunstein and Martha C Nussbaum (eds), *Animal Rights: Current Debates and New Directions* (OUP 2005); Peters (n 2) 47–8.

111 See Stone (n 110) 458ff; Tribe (n 97) 3.

112 See eg Constitutional Court of South Africa 8 December 2016, CCT 1/16 (affirming the National Council of Societies for the Prevention of Cruelty to Animals’ statutory power of private prosecution and to institute legal proceedings in case of animal cruelty offences).

113 See Frederick Schauer, ‘Proportionality and the Question of Weight’ in Grant Huscroft, Bradley W Miller and Grégoire Webber (eds), *Proportionality and the Rule of Law: Rights, Justification, Reasoning* (CUP 2014) 177–8.

114 See generally Saskia Stucki, *Grundrechte für Tiere* (Nomos 2016) 151ff.

115 For example, under the Swiss 2005 Animal Welfare Act (Tierschutzgesetz), life itself is not a legally protected good, and the (painless, non-arbitrary) killing of an animal does not therefore require any justification.

116 See also *Noah v Attorney General* (n 60) 253–4 (pointing out that balancing different interests is ‘part and parcel of our legal system’).

117 See generally Edmundson, ‘Do Animals Need Rights?’ (n 2) 346; Sunstein, ‘Rights and Their Critics’ (n 65) 736–7.

118 On this threshold-raising conception of rights, see generally Schauer, ‘A Comment on the Structure of Rights’ (n 85) 430; Ronald Dworkin, *Taking Rights Seriously* (Harvard UP 1978) 191–2 (noting that a right cannot justifiably be overridden ‘on the minimal grounds that would be sufficient if no such right existed’).

119 At present, the overwhelming portion of permissible interferences with animals’ interests can hardly be said to be necessary or proportionate in any real sense of the word. See Francione, *Introduction to Animal Rights* (n 17) 9, 55.

120 As noted by Teubner, animal rights ‘create basically defensive institutions. Paradoxically, they incorporate animals into human society in order to create defences against the destructive tendencies of human society against animals’. Gunther Teubner, ‘Rights of Non-Humans? Electronic Agents and Animals as New Actors in Politics and Law’ (2006) 33 Journal of Law and Society 497, 521.

121 See eg Mark Tushnet, ‘An Essay on Rights’ (1984) 62 Tex L Rev 1363; Mary Ann Glendon, *Rights Talk: The Impoverishment of Political Discourse* (Free Press 1991); for a modern reformulation of the rights critique, see eg Robin L West, ‘Tragic Rights: The Rights Critique in the Age of Obama’ (2011) 53 Wm & Mary L Rev 713.

122 See generally Alan Dershowitz, *Rights from Wrongs: A Secular Theory of the Origins of Rights* (Basic Books 2004) 59ff.

123 See Sunstein, ‘Rights and Their Critics’ (n 65) 754.

124 Dershowitz (n 122) 9.

125 Jeremy Waldron, ‘When Justice Replaces Affection: The Need for Rights’ (1988) 11 Harv JL & Pub Pol’y 625, 629.

126 See Edmundson, ‘Do Animals Need Rights?’ (n 2) 358.

127 More generally, the practical need for rights as complementary or compensatory guarantees will vary depending on social context, and may be more immediate and pressing for the disempowered, disenfranchised, marginalised, victimised, vulnerable, disadvantaged or even oppressed portions of society. See generally Patricia J Williams, ‘Alchemical Notes: Reconstructing Ideals from Deconstructed Rights’ (1987) 22 Harvard Civil Rights-Civil Liberties Law Review 401.

128 Donaldson and Kymlicka (n 1) 40, 49; see further Tom Regan, *The Case for Animal Rights* (University of California Press 2004) 330ff, 348–9; Bilchitz, ‘Moving Beyond Arbitrariness’ (n 34) 69.

129 See Bilchitz, ‘Moving Beyond Arbitrariness’ (n 34) 69.

130 On the aspirational dimension of human rights, see generally Philip Harvey, ‘Aspirational Law’ (2004) 52 Buff L Rev 701.

131 ibid 717–18; Raz, ‘Legal Rights’ (n 49) 14–15, 19; ‘rights are to law what conscious commitments are to the psyche’. Williams (n 127) 424.

132 See David Bilchitz, ‘Fundamental Rights as Bridging Concepts: Straddling the Boundary Between Ideal Justice and an Imperfect Reality’ (2018) 40 Hum Rts Q 119, 121ff.

133 Donaldson and Kymlicka (n 1) 49; see also Gary L Francione, *Rain Without Thunder: The Ideology of the Animal Rights Movement* (Temple UP 2007) 2.

134 cf Kymlicka and Donaldson (n 12) 331–2.

135 On the dynamic nature of rights and their generative power, see Raz, *The Morality of Freedom* (n 50) 171; Waldron, ‘Rights in Conflict’ (n 86) 212, 214.

136 See David Bilchitz, ‘Does Transformative Constitutionalism Require the Recognition of Animal Rights?’ (2010) 25 Southern African Public Law 267, 291ff.

137 Bilchitz, ‘Moving Beyond Arbitrariness’ (n 34) 71.

138 cf Harvey (n 130) 723 (noting that human rights will always remain a ‘work in progress rather than a finished project’); similarly, Kymlicka and Donaldson (n 12) 333.

139 Stone (n 110) 453.

## Notes

This article is the first part of my postdoctoral research project ‘Trilogy on a Legal Theory of Animal Rights’, funded by the Swiss National Science Foundation. For helpful comments on earlier versions of this article, I am indebted to William Edmundson, Raffael Fasel, Chris Green, Christoph Krenn, Visa Kurki, Will Kymlicka, Nico Müller, Anne Peters, Kristen Stilt, MH Tse, Steven White, Derek Williams and the anonymous reviewers for the Oxford Journal of Legal Studies.

